# Connections and Unmet Needs: Severe Asthma Biologics and Osteoporosis

**DOI:** 10.3390/biomedicines13010197

**Published:** 2025-01-15

**Authors:** Fabiana Furci, Marco Umberto Scaramozzino, Giuseppe Rocco Talesa, Corrado Pelaia

**Affiliations:** 1 Provincial Healthcare Unit, Section of Allergology, 89900 Vibo Valentia, Italy; fabianafurci@gmail.com; 2 “La Madonnina” Outpatient Clinic, 89100 Reggio Calabria, Italy; 3 Department of Orthopaedics and Traumatology, San Matteo Degli Infermi Hospital, 06049 Spoleto, Italy; talesa.giuseppe@libero.it; 4 Department of Medical and Surgical Sciences, University of Magna Graecia of Catanzaro, 88100 Catanzaro, Italy; pelaia.corrado@gmail.com

**Keywords:** severe asthma, biologics, corticosteroids, inflammation, interleukins, osteoporosis

## Abstract

Asthma is a chronic inflammatory disease with the main anti-inflammatory drugs for better disease control being steroids or corticosteroids. The use of steroids in asthma patients, in particular in uncontrolled asthma patients, is associated with an increased risk of osteoporosis and fragility fractures. A single oral corticosteroid course increases the risk of osteoporosis and the continual use of inhaled corticosteroids is correlated over time to an increased risk for both bone conditions. With the use of new, available biologic therapies for asthma, perhaps even anticipating the times of their use in therapeutic management, in the current guidelines and with targeted strategies of prevention it may be possible to improve asthma management, preventing some comorbidities, such as osteoporosis.

## 1. Introduction

Asthma and osteoporosis are two medical conditions that may appear distinct, but which are interconnected through a series of pathophysiological mechanisms and shared risk factors. This article explores the links between asthma and osteoporosis, focusing on how chronic inflammation, glucocorticoid use, and other common risk factors contribute to the development of both diseases. Additionally, therapeutic implications and management strategies for patients suffering from both conditions are discussed. Asthma is a chronic inflammatory disease of the airways characterized by reversible bronchial obstruction, bronchial hyperreactivity, and the recurring symptoms of wheezing, dyspnoea, chest tightness, and cough. Asthma management is based on international guidelines that include the use of bronchodilators and inhaled corticosteroids (ICSs) to control inflammation and prevent exacerbations [1]. Osteoporosis is a systemic skeletal disease characterized by reduced bone mass and the deterioration of bone microarchitecture, leading to increased bone fragility and fracture risk. The risk factors for osteoporosis include advanced age, menopause, calcium and vitamin D deficiency, and prolonged use of glucocorticoids [2]. Chronic inflammation present in asthma can negatively impact bone health. Pro-inflammatory cytokines such as IL-6 and TNF-α, which are elevated in asthma, can stimulate bone resorption, reducing bone mineral density (BMD) [3]. Glucocorticoids, frequently used in asthma treatment, are well known for their adverse effects on bone mass. Chronic use of glucocorticoids can decrease intestinal calcium absorption and inhibit bone formation, significantly increasing the risk of osteoporosis and fractures [4]. Studies have shown that both oral corticosteroids (OCSs) and ICSs can contribute to bone loss, although the effects are more pronounced with OCSs [5,6], which accelerate bone loss and hence, increase the risk of fracture in patients taking > 7.5 mg of prednisolone daily [7]. However, evidence highlights that low- and medium-dose ICSs are safe and have little to no effect on BMD and metabolism [8].

A sedentary lifestyle is a risk factor for both conditions. Regular physical activity, particularly weight-bearing and resistance exercises, can improve both pulmonary function and bone health [9]. A diet poor in calcium and vitamin D can contribute to osteoporosis and worsen general health, negatively affecting asthma management. Proper nutrition is essential for maintaining good bone health and preventing chronic inflammation [10]. Some genetic factors may predispose individuals to both conditions. Ongoing research continues to explore the genetic links between asthma and osteoporosis, aiming to identify common genes that may influence the development of these diseases [11]. Asthma management should include the prudent use of glucocorticoids, balancing respiratory benefits with the risk of osteoporosis. Using inhalers at the minimal effective dose and introducing alternative medications where possible can help mitigate this risk. Additionally, the use of drugs such as bisphosphonates may be indicated to prevent bone loss in patients requiring long-term glucocorticoids [12]. For asthma patients using glucocorticoids, supplementation with calcium and vitamin D may be indicated. This approach can help maintain BMD and reduce the risk of osteoporosis. Tailored exercise programs will help. Exercise not only helps maintain good bone health but can also improve asthma control, reducing the frequency and severity of exacerbations.

**Figure 1:**
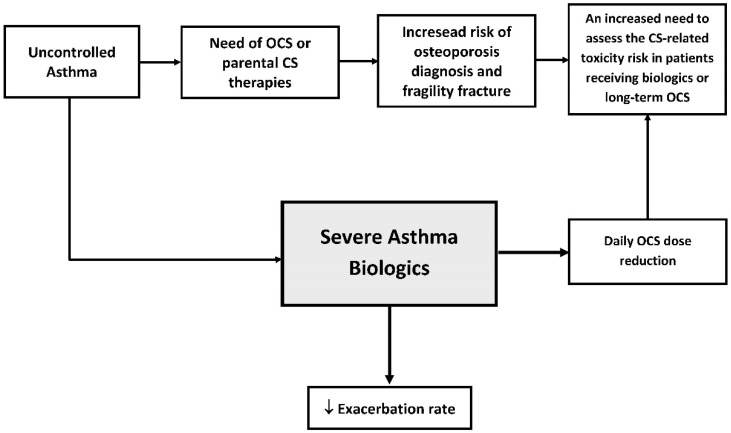
Flow chart for severe asthma management.

The use of biological therapies has revolutionized the treatment of severe asthma. These therapies target specific pathways involved in the inflammatory process, offering a more personalized approach to asthma management. Biologics such as IL-5 and IL-5R (mepolizumab, reslizumab, and benralizumab) and the IL-4 receptor subunit α of IL-4 shared by the IL-4 and IL-13 receptor complexes, thereby inhibiting IL-4 and IL-13 signaling (dupilumab), have shown significant efficacy in reducing asthma exacerbations, improving lung function, and enhancing the quality of life (QoL) for patients with severe asthma. The introduction of these biologics also has implications for bone health. By potentially reducing the need for systemic glucocorticoids, biologics may help mitigate the associated risk of osteoporosis [13] (Figure 1). Considering that asthma patients exposed to OCSs or high-dose ICSs are more susceptible to bone comorbidities, asthma biologics have reported a safer profile, which, together with the efficacy of biologics, can make corticosteroids (CSs) the last therapeutic choice after biologics. As reported by Domingo C. et al., asthma biologics, such as mepolizumab, induce a de-escalation effect in OCS maintenance dose and a reduction in cumulative CS exposure in many severe asthma patients [14].

In particular, the reduction or avoidance of chronic OCS use in asthma management correlates with a reduction in the risk of osteoporosis and fractures [3,14]. Indeed, osteoporosis prevention treatment is recommended for patients (with additional risk factors) aged < 40–50 years in treatment with prednisolone > 7.5 mg/day or aged > 50 years receiving ≥ 5 mg/day [15]. Between January 2015 and February 2021, in a propensity score-matched, prospective cohort study using data from the International Severe Asthma Registry in which severe asthma patients and high OCS (HOCS) exposure (long-term OCSs for ≥1 year or ≥4 courses of rescue OCSs within a 12-month period) were identified, the initiation of biologics reported improvements in asthma outcomes, such as exacerbation rate, OCS exposure, and healthcare resource utilization. Patients treated with biologics had twice as high a chance of achieving a daily long-term OCS dose of less than 5 mg and a four times higher chance of reducing their total OCS dose by more than 75% from baseline than patients not treated with severe asthma biologics [16].

As reported by Chalictsios VV. et al., asthma patients treated with OCS or high ICS doses have a higher risk of presenting bone comorbidities and, therefore, striking the right balance between the efficacy and safety of steroids in these patients is important to improve QoL.

The use of glucocorticoids can induce osteoporosis, the most common adverse effect of corticosteroids. Osteoporosis is characterized by the structural deterioration of bone tissue and low bone mass, with consequent bone fragility and increased risk of fracture. Decreased BMD is related to a higher fracture risk. Fractures are related to morbidity, mortality, and increased healthcare costs [3]. Thus, the pharmacoeconomic impact of biological therapies in asthma management is substantial. While these treatments are often more expensive upfront compared to traditional therapies, they can reduce healthcare costs in the long term by decreasing the frequency of severe asthma exacerbations, hospitalizations, and the overall burden of the disease. Furthermore, by potentially reducing the need for systemic glucocorticoids, biologics may lower the incidence of glucocorticoid-induced osteoporosis, thereby reducing the costs related to osteoporosis management and fracture treatment [17]. It is crucial to regularly monitor BMD in asthmatic patients undergoing long-term glucocorticoid therapy. This can be carried out through exams such as dual-energy X-ray absorptiometry (DEXA), which allows the early identification of bone loss and intervention with preventive measures. The integrated management of asthma and osteoporosis requires a multidisciplinary approach that considers the interconnection between the two conditions. A thorough understanding of the common mechanisms and preventive strategies can improve the QoL for patients affected by both diseases. Collaboration between pulmonologists, endocrinologists, and other specialists is essential to develop personalized treatment plans that address the specific needs of patients. Ultimately, the management of asthma and osteoporosis must consider the interplay between these conditions. Ensuring optimal asthma control while minimizing the adverse effects of treatment on bone health requires careful consideration of medication choices, lifestyle modifications, and regular monitoring. Reviewing the CS dose and using the lowest dose possible is a key strategy in asthma management [4]. The advent of biological therapies, considered as OCS-sparing agents, provides new opportunities for managing severe asthma more effectively and with potentially fewer side effects, highlighting the importance of ongoing research and innovation in the treatment of these chronic conditions.

## 2. Materials and Methods

### 2.1. Osteoporosis and the IL-31/33 Axis

**Figure 2:**
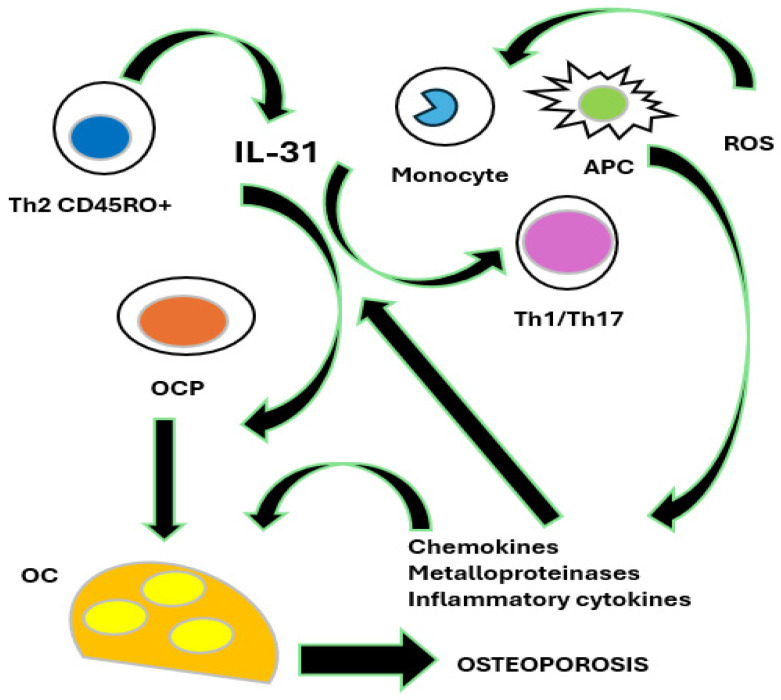
The role of IL-31 in bone remodeling. IL-31, mainly produced by T helper 2 memory cells (Th2 CD45RO+), promotes osteoclastogenesis by inducing the differentiation of osteoclast progenitors (OCPs) into mature osteoclasts (OCs). IL-31, in synergy with reactive oxygen species (ROS), also stimulates osteoclastogenesis indirectly by inducing antigen-presenting cells (APCs), monocytes, and T helper 1 and 17 lymphocytes (Th1/Th17) to produce chemokines, metalloproteinases, and inflammatory cytokines, which in turn increase the production of IL-31. All of these events lead to the development of osteoporosis.

**Figure 3:**
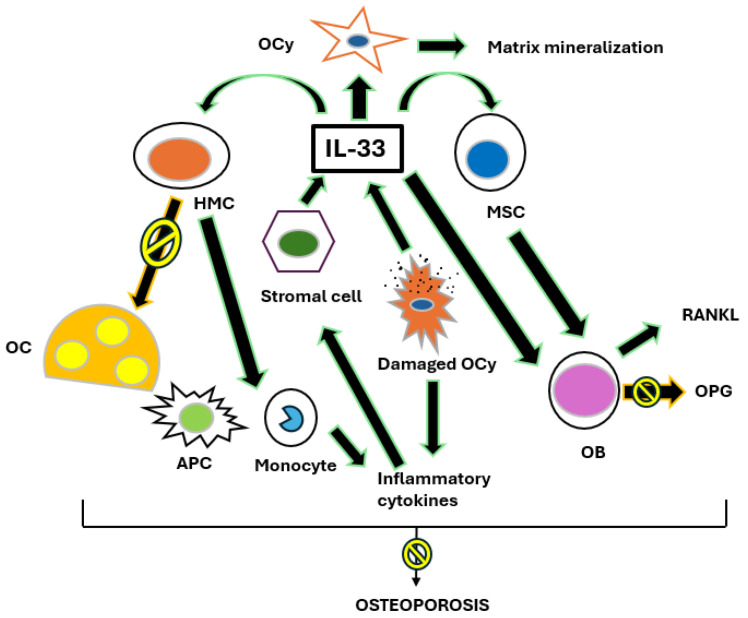
The role of IL-33 in bone remodeling. After inflammatory stimulation, the stromal cells produce IL-33, which directly blocks osteoclast (OC) formation from hemopoietic myeloid cells (HMCs), shifting the osteoclast precursor differentiation towards cytokine-producing macrophages and antigen-presenting cells (APCs). Damaged osteocytes (OCys), in addition to inducing inflammation, also release IL-33, which in turn stimulates the mineralization of the bone matrix. IL-33 acts directly by stimulating mature osteoblasts (OBs) and their bone marrow progenitors, the mesenchymal stem cells (MSCs), inducing their differentiation and maturation. Despite the production of the receptor activator of nuclear factor kappa-Β ligand (RANKL) and the partial blockage of the production of osteoprotegerin (OPG) by activated osteoblasts, the prevalent effect of IL-33 on the skeleton is anti-osteoporotic. Arrows with a “forbidden” signal mean that il-33 has anti-osteoporotic activities.

Bronchial asthma is a chronic respiratory disease characterized by the inflammation and hyperreactivity of the airways. The interleukins IL-31 and IL-33 play a crucial role in the pathological mechanisms that govern this condition [4]. IL-31, produced mainly by T helper 2 (Th2) cells, is involved in modulating the inflammatory response. This cytokine is known to promote the activation of mast cell and basophil cells, facilitating allergic inflammation and contributing to the production of other pro-inflammatory cytokines and chemokines, which in turn amplify asthma symptoms [17,18,19]. Its secretion is increased in conditions of severe asthma, suggesting a direct link between elevated IL-31 levels and disease severity [20]. On the other hand, IL-33, a high alarmin released by damaged epithelial cells, plays a critical role in the activation of immune system cells, including T cells and mast cells [21]. Activation occurs in situations of environmental stress or tissue injury, stimulating a robust inflammatory response. IL-33 induces the production of other Th2 cytokines, such as IL-4 and IL-13, which are important for the allergic response and chronic inflammation in the airways. Interactions between IL-31 and IL-33 lead to further amplification of inflammation, contributing to the pathogenesis of asthma [22]. This chronic inflammation is not only limited to the airways, but also causes systemic effects that can affect bone metabolism. Pro-inflammatory cytokines, activated by the mediators of the IL-31/IL-33 axis, can stimulate osteoclastogenesis, i.e., the formation of osteoclasts, cells responsible for bone resorption. As a result, asthma patients with sustained inflammation may be at increased risk of developing osteoporosis [23]. Furthermore, prolonged use of CS for asthma control further compromises BMD, making patients vulnerable to bone fractures [24]. Therefore, it is critical for pulmonary physicians to integrate an osteoporosis risk assessment into the management of asthma patients, as chronic inflammation and CS treatment may interact negatively to impact bone health. The identification of the IL-31/IL-33 axis as a risk factor for osteoporosis not only improves the understanding of the pathophysiology of asthma, but also highlights the importance of therapeutic strategies that consider both respiratory and systemic symptoms. Preventive strategies such as the adoption of a healthy lifestyle, combined with scrupulous pharmacological management, can have their say in mitigating the side effects linked to osteoporosis in these patients [6,25]. Continued research on the effects of IL-31 and IL-33 in asthma may lead to new therapeutic innovations, thus improving the QoL of asthma patients and reducing the risk of associated complications (Figure 2 and Figure 3) [26].

### 2.2. Overview of Available OCS and Conversion of Doses of OCS

The main treatment in asthma is the use of CS given their capacity to act on inflammation which is typical of asthma. It is important to understand the correct use of the available corticosteroids and their pharmacokinetics. An OCS conversion table can be found at the end of the paragraph (Table 1) [27].

Cortisone: This hormone acts on the intermediate metabolism, increasing the availability of glucose, and inducing proteolysis and lipolysis, thus placing the organism in an energetically active state, which is useful for overcoming many stressful conditions; it alters the normal fluid and electrolyte balance, increasing the reabsorption of sodium and the excretion of potassium and calcium. Finally, it induces vasoconstriction at the microcirculation level. Indicated in the treatment of acute and chronic inflammatory pathologies that require systemic therapies with OCS, it can also be useful in the management of symptoms in allergic and neoplastic conditions [28].

Hydrocortisone: Hydrocortisone is the main glucocorticoid produced by the adrenal cortex. It works by binding to its receptors present inside the cells, promoting the expression of some specific genes with the consequent activation of molecules with anti-inflammatory action and the simultaneous inhibition of molecules with pro-inflammatory activity. It is used against inflammation, allergic reactions, diseases involving collagen, asthma, adrenal insufficiency, some forms of cancer, Addison’s disease, and autoimmune diseases such as arthritis, lupus, psoriasis, ulcerative colitis, and Crohn’s disease [29].

Deflazacort: This is a corticosteroid which, after oral intake, is absorbed rapidly and completely in the intestine with peak plasma levels reached in 1–2 h. Subsequently, the original compound is deacetylated at position 21 to be transformed into an active metabolite characterized by high binding affinity for tissue glucocorticoid receptors. The absolute oral bioavailability is 68%, the binding to plasma proteins is 39.8%, while the almost complete elimination of the active metabolite occurs within 24 h mainly through urine. It is a drug that has indications in the treatment of various pathologies including sarcoidosis, juvenile chronic arthritis, polymyalgia rheumatica, and rheumatoid arthritis [30].

Prednisolone: This works by preventing the release of molecules that trigger inflammation. It is used in the treatment of many different disorders associated with inflammation, for example, allergies, ulcerative colitis, arthritis, lupus, psoriasis, or respiratory problems [31].

Prednisone: This is a synthetic hormone that belongs to the corticosteroid group, and has properties to reduce pain, swelling, stiffness, redness and heat in affected tissues. It is used in some rheumatological conditions such as rheumatoid arthritis, acute gouty arthritis, Still’s disease, ankylosing spondylitis, systemic lupus erythematosus, dermatomyositis, bronchial asthma, atopic and contact dermatitis, in sarcoidosis, to treat some diseases of the blood, as palliative therapy for some tumors, in addition to therapies for gastrointestinal pathologies [32].

Methylprednisolone: Methylprednisolone is a powerful anti-inflammatory that, similarly to other corticosteroid drugs, appears to inhibit the release of arachidonic acid, the precursor of important inflammatory mediators. Oral methylprednisolone is used in the treatment of adrenal insufficiency; joint pathologies, such as psoriatic arthritis and rheumatoid arthritis; skin conditions such as severe psoriasis, pemphigus, allergic diseases; eye disorders such as diffuse posterior uveitis, optic neuritis, iritis, and iridocyclitis; lung diseases such as sarcoidosis and emphysema; blood pathologies and tumors such as leukemia; intestinal diseases such as ulcerative colitis and regional enteritis; brain disorders (e.g., tuberculous meningitis); and other pathologies [33].

Triamcinolone: This acts from a molecular point of view, determining the expression of the lipocortin enzyme, capable of inhibiting phospholipase A2 and consequently reducing the availability of arachidonic acid. The reduced concentration of the starting substrate translates into a modest synthesis of inflammatory mediators such as leukotrienes, prostaglandins, and prostacyclins with the consequent control of the entire inflammatory process and related tissue damage. Once its activity is complete, the active ingredient is metabolized in the liver, mainly through hydroxylation processes, and subsequently excreted via the kidneys. It can be used to treat many different conditions: allergies, disorders of the skin and related tissues (hair and nails) such as psoriasis, rheumatic diseases, ulcerative colitis (chronic inflammatory bowel disease), and some respiratory diseases [34].

Betamethasone: This belongs to the class of long-acting glucocorticoids. The main indications are for the treatment of bronchial asthma, severe allergic diseases, rheumatoid arthritis, collagenopathies, inflammatory skin diseases, tumors of the lymphatic system such as acute and chronic malignant hemolymphopathies, Hodgkin’s disease, nephrotic syndrome, ulcerative colitis and Crohn’s disease, pemphigus, sarcoidosis, rheumatic carditis, ankylosing spondylitis, hemolytic anemia, agranulocytosis, and thrombocytopenic purpura [35].

Dexamethasone: This is a synthetic hormone with an anti-inflammatory activity approximately 7 times more powerful than prednisolone and 30 times more powerful than hydrocortisone. The molecule exerts its action on the hypothalamic–pituitary–adrenal axis by binding to specific receptors on the cellular plasma membrane. In other tissues, the molecule passes cell membranes and diffuses into the cytosol, combining with specific cytoplasmic receptors, the glucocorticoid receptor. The complex that is formed enters the cell nucleus and stimulates protein synthesis. Like other adrenocortical steroids, dexamethasone has anti-allergic, anti-shock, antitoxic, antifebrile, and immunosuppressive properties. Characteristically, this molecule predominantly presents glucocorticoid activity; its ability to determine the renal retention of sodium and water (i.e., mineralocorticoid activity) is vastly lower than that of other molecules of the same class. After oral administration, dexamethasone is rapidly absorbed from the gastrointestinal tract. Following intravenous injection, peak plasma levels are reached in 5 min. Its half-life in plasma is approximately 190 min. It may be more interesting to consider the biological half-life of the molecule, which is equal to 36–54 h, which makes dexamethasone a substance that is indicated for the treatment of pathologies that require prolonged glucocorticoid action such as in patients suffering from rheumatoid arthritis, bronchial asthma, purpura, undergoing chemotherapy, etc. [36].

### 2.3. The Impact of Severe Asthma Biologics in Sparing Steroid Therapy

Severe asthma biologics that act on the different steps of type 2 inflammation are a revolutionary strategy to treat the disease. When the diagnosis of asthma is confirmed and comorbidities have been addressed, such as rhinitis, rhinosinusitis, nasal polyps, and gastroesophageal reflux, severe asthma is classified as “asthma which requires treatment with high dose ICSs plus a second controller (and/or systemic corticosteroids) to prevent it from becoming ‘uncontrolled’ or which remains ‘uncontrolled’ despite this therapy.” Patients affected by uncontrolled asthma present high rates of exacerbations with the need for glucocorticoid therapy.

There are now several mAb therapies (“biologics”) available to treat severe asthma. These drugs all reduce the exacerbation rate [37]. Once comorbidities have been addressed, and excluding those patients who are poorly adherent to inhaled therapy, the prevalence of severe asthma affects approximately 3.7% of the asthma population [1], resulting in significant asthma-related healthcare costs, frequent exacerbations, impairment of QoL, missed work/school days, and frequent healthcare utilization. Severe asthma exacerbations need OCS or may need maintenance oral corticosteroids (mOCSs) to control the disease. The long-term use of OCS is associated with significant long-term morbidity, such as adrenal suppression, osteopenia and osteoporosis, increased risk of type II diabetes, cataracts, and obesity [38]. Asthma can be considered the result of the combination of genetic susceptibility with external factors such as allergens, microbes, pollutants, and other triggers that, acting on the airway epithelium, induce the release of “alarmins”, including interleukin (IL)-25, IL-33, and thymic stromal lymphopoietin (TSLP), which result in the production of T2 cytokines from cells including Th2 cells and type 2 innate lymphoid cells (ILC2) [39]. Severe asthma biologics that are essential in the exacerbation pathogenesis of asthma act on one or more mediators or cells within this pathway. Type 2 cytokines that play a key role in asthma disease include IL-4, related to IgE production from B-cell;, IL-5, related to the eosinophil cycle; and IL-13, related to mucus hypersecretion and airway hyperresponsiveness [40].

Omalizumab (“Xolair”), the first mAb approved to treat severe asthma, with an antiviral response and a reduction in virus-induced exacerbations, binds to free circulating IgE, inducing the inhibition of attachment to its receptor (FCεRI) and a reduction in downstream effects, including mast cell degranulation and the expression of inflammatory cytokines such as IL-3, IL-4, IL-5, IL-6, and IL-13 [41]. Various real-world, observational trials reported mOCS dose reduction in patients treated with omalizumab [42,43,44,45]. In a systematic review of 42 observational or registry trials, the authors highlighted a mean OCS dose reduction of 68% at 12 months, with a broad range from −78% to −12% [46]. The large “eXpeRience” registry reported on over 260 patients on baseline OCS and reported that 57% either reduced or stopped mOCS with 1 year of omalizumab treatment. Moreover, it was reported that the mean prednisolone dose at the end of 1 year was 7.7 mg compared to 15.5 mg at baseline [47]. In a randomized trial, a major reduction or interruption of mOCS in patients treated with omalizumab rather than with optimal standard care was described [48].

Mepolizumab (“Nucala”), the first mAb introduced for severe eosinophilic asthma, acts on the IL-5 cytokine involved in the development, migration, and survival of eosinophils [38,40]. The phase 3 MENSA trial reported a reduction in exacerbations (52%) in patients treated with mepolizumab compared to patients treated with placebo [47] and, moreover, a significant reduction in OCS dose in patients dependent on daily OCS to maintain asthma control. The SIRIUS trial reported that at 24 weeks, 14% of the asthma patients in mepolizumab treatment were able to stop prednisolone with a general median reduction of 50% OCS use [48].

A retrospective analysis of 99 patients in mepolizumab treatment reported a 50% reduction in exacerbation rate, with a reduction in prednisolone dose of at least 50% [49]. Regarding Reslizumab (“Cinqaero”), a recombinant humanized IgG4 mAb that acts on IL-5, efficacy in terms of reduction in mOCS use has not been formally reported. Indeed, mepolizumab, benralizumab, and dupilumab are the only severe asthma biologics for which a significant reduction in daily OCS use has been reported.

Benralizumab (“Fasenra”), which acts on the IL-5 pathway through ligation to the α subunit of the IL-5 receptor (IL-5R-α), induces cell-mediated cytotoxicity and consequent cell apoptosis (eosinophils and basophils) on which IL-5R-α is expressed [50,51]. In the ZONDA study, which enrolled OCS-dependent asthma patients, a 50% reduction in OCS dose in benralizumab-treated patients was reported compared with placebo. Moreover, a reduction of 70% in exacerbations was described [52]. In the open-label PONENTE study, it was reported that in 598 OCS-dependent patients treated with benralizumab over 80% were able to stop the use of steroids, or achieved a dosage of 5 mg or less if the reason for stopping the reduction was adrenal insufficiency rather than asthma [53].

Dupilumab (“Dupixent”), an mAb targeted against the α subunit of the IL-4 receptor, has also reported a significant reduction in mOCS use in asthma. The LIBERTY ASTHMA VENTURE trial reported an overall median prednisolone dose reduction of 50% compared to placebo, and 69% reduced their dose below 5 mg·day 1 compared with 33% of the placebo group [54]. Moreover, in this trial, a significant reduction in corticosteroid dose in both low (<300 cells·μL^−1^) and high (≥300 cells·μL^−1^) blood eosinophil groups was seen, although the greatest response was reported in the asthma patients with higher baseline blood eosinophil counts [55].

Tezepelumab is an mAb that targets the epithelial alarmin TSLP, which is released by epithelial cells in response to various pro-inflammatory stimuli and related to both T2 cytokine expression and asthma severity in asthma [56,57]. In the SOURCE trial, with patients with OCS-dependent asthma, randomized to tezepelumab or placebo, the investigators reported that the trial did not meet the primary endpoint of reducing daily OCS whilst maintaining asthma control [58,59].

## 3. Discussion

Asthma patients are characterized by an increased risk of osteoporosis and fragility fractures, in particular of vertebra and forearm/wrist fractures, compared with the general population. The mechanism of risk for osteoporosis in asthma patients can be divided into asthma-induced osteoporosis and corticosteroid-induced osteoporosis. The direct effect of asthma leading to osteoporosis is related to disease inflammation (e.g., neutrophil-mediated inflammation, TNF-α and IL-6). TNF-α, an inhibitor of osteogenesis and an activator of osteoclastogenesis, is involved in neutrophil migration, which is associated with a decrease in BMD. IL-6 is also an important inflammatory factor involved in neutrophil-inflammation-mediated osteoporosis in asthma patients. The overexpression of IL-6 induces an inhibition of the differentiation of osteoblasts, with an increased expression of RANKL and an increase in osteoclast activity [60].

Knowledge of the risk of CS-related toxicities correlated to OCS or parenteral CS therapies for the management of acute severe asthma exacerbations is an important factor to consider in terms of asthma management and safety. It has been reported that the risk of osteoporosis diagnosis and fracture increases per 1 g in systemic CS exposure in asthma patients [38]. Therefore, the use of anti-osteoporotic treatment is essential in adult patients with high cumulative CS exposure (>5 g/year) [15,61]. Moreover, an analysis published in 2021 highlighted how there is an inter-person variability in the risk of OCS-related toxicity among severe asthma patients, as measured by the Glucocorticoid Toxicity Index (GTI), with only a modest correlation between recent OCS exposure and GTI score [62]. This inter-variability appears to be part of the heterogeneity of patients suffering from asthma, with their different phenotypes and endotypes and varying responses to treatments, including biological therapies, which have shown a more favorable safety profile compared to therapies that correlate to multi-comorbidities [62,63]. Indeed, as reported by Cutroneo PM et al. conducting an overview of the safety data of severe asthma biologics in VigiBase, the World Health Organization global pharmacovigilance database, the most frequently reported suspected adverse drug reactions are the well-known adverse effects such as general disorders, injection-site reactions, nasopharyngitis, headache, and hypersensitivity, while others, such as asthma exacerbation or therapeutic failure, may be related to the indication of use. The authors reported that a significant confounder in the analysis conducted is represented by the concomitant or recent use of OCSs by severe asthma patients [64]. Albeit, after starting biologics, OCS sparing up to withdrawal can be considered a primary outcome in the management of severe asthma patients to minimize side effects, and the frequent or continuous use of OCSs or long-term high-dose ICSs could be associated with many potential adverse events, such as fluid retention, bone damage, elevated blood sugars, and psychiatric problems. These could be clinically relevant, in particular when high doses are administered for a prolonged period of time [65]. The reduction or suppression of OCSs during severe asthma biological therapy may unmask some comorbidity symptoms or patients’ underlying diseases (e.g., psoriasis and multiple inflammatory diseases), known as glucocorticoid deprivation syndrome [64].

From the knowledge of the molecules involved in asthmatic inflammation, such as IL-6, it is possible to understand the pivotal role of severe asthma biological therapies both in terms of asthma and in relation to pathologies such as osteoporosis which correlate with asthma inflammation [14,66]. Indeed, as reported by Perlato M. et al., considering that in severe asthma patients, apart from the phenomenon known as “airway remodeling”, the prolonged use of OCS induces “patient remodeling”, with the manifestation of physical changes (e.g., suppression of hypothalamic–pituitary–adrenal axis, diabetes, adrenal insufficiency, obesity, buffalo hump, striae rubrae, and osteoporosis), the use of biologics can improve asthma control and avoid or limit the inflammation-induced “airway remodeling” and the OCS-induced “patient remodeling” [67].

The core of the multidisciplinary approach to airway diseases is a person-centered model of care focused on the “Treatable Traits” that are causing symptoms and impacts. This approach is integral to addressing the complexity and heterogeneity of severe asthma patients [68].

In severe asthma, multidisciplinary care that includes diverse healthcare professionals (e.g., endocrinologists, allergists, otolaryngologists, and gastroenterologists) that involves systematic or multidimensional assessment is recommended by international guidelines [37,69]. Observational and registry studies, and a meta-analysis, reported that these approaches to severe asthma management induce an improvement in asthma control (SMD 0.36 (95% CI 0.23–0.59)) and in asthma QoL (SMD 0.36 (95% CI 0.22–0.50)), reduce acute attacks (SMD −0.34 (95% CI −0.44– −0.23)), and reduce OCS use (reduction of 11.5 mg on average) [70,71].

## 4. Conclusions

Physicians managing severe asthma should remain vigilant in assessing the CS-related toxicity risk in patients receiving biologics or long-term OCS, especially in patients with additional osteoporotic risk factors. A timely diagnosis is necessary in order to increase the possibility of having important comorbidities following prolonged use of oral steroids and reduce the costs of healthcare spending due to excessive use of OCS and hospitalization of patients suffering from severe asthma.

## Figures and Tables

**Table 1:** Corticosteroid conversion table drawn up from the available literature with clinical studies of sufficiently homogeneous enrolled populations.

Oral Corticosteroids (OCS)	Approximate Equivalent Dose (mg)
Cortisone	25 mg
Hydrocortisone	20 mg
Deflazacort	7.5 mg
Prednisolone	5 mg
Prednisone	5 mg
Methylprednisolone	4 mg
Triamcinolone	4 mg
Betamethasone	0.75 mg
Dexamethasone	0.75 mg

## Data Availability

Data are contained within the article.
